# Modelling the challenges of managing free-ranging dog populations

**DOI:** 10.1038/s41598-020-75828-6

**Published:** 2020-11-02

**Authors:** Aniruddha Belsare, Abi Tamim Vanak

**Affiliations:** 1 Department of Fisheries and Wildlife, Boone and Crockett Quantitative Wildlife Center, Michigan State University, East Lansing, MI USA; 2 OneHealth Working Group, Center for Modeling Complex Interactions, University of Idaho, Moscow, ID USA; 3 Ashoka Trust for Research in Ecology and the Environment, Bangalore, India; 4 DBT/ Wellcome Trust, India Alliance Program (Clinical and Public Health Fellowship), Hyderabad, India; 5 School of Life Sciences, University of KwaZulu-Natal, Durban, South Africa

**Keywords:** Viral infection, Ecological modelling, Population dynamics

## Abstract

Free-ranging domestic dogs (FRD) are not only vectors of zoonoses of public health concern, but also pose direct threats to humans, livestock, and endangered wildlife. Many developing countries have struggled to control FRD, despite using both lethal and non-lethal methods. India has amongst the highest FRD populations globally and the highest incidences of dog-mediated human rabies, but only deploys Catch–Neuter–Vaccinate–Release (CNVR) for FRD control as a humane alternative to lethal methods, without evidence of it working successfully. Here, we use an agent-based dog population dynamics model to examine the time, effort, financial resources, and conditions needed to successfully control FRD in a typical urban setting. We simulate several scenarios, from an “ideal world” closed population with easily accessible dogs, to a more realistic open population with heterogeneity in catchability of dogs. In only one “best-case” scenario, CNVR resulted in a significant and lasting reduction in FRD, but with vaccination rates peaking only at 35%, which is half the WHO-recommended coverage. The customisable and portable modelling tool that we have developed allows managers to simulate real world processes and understand the expected effort needed to reduce regional dog populations, and assess methods for achieving effective anti-rabies vaccination coverage.

## Introduction

One of the most common terrestrial carnivores in the world, the domestic dog (*Canis familiaris*) is found on every continent that humans have settled^1^. More than 70% of the global dog population (estimated at > 700 million to ~ 1 billion) comprises of free-ranging dogs (FRD)^1,2^. In many developing countries, FRD are associated with the transmission of zoonotic diseases such as rabies, zoonotic visceral leishmaniasis, canine echinococcosis, and soil borne helminths^3–7^. Rabies alone is responsible for an estimated 60,000 human deaths per year worldwide, with a majority of these deaths occurring in Asia and Africa^8^. In addition, FRD are an important and emerging threat for livestock^9,10^ as well as biodiversity^2,11–14^. Furthermore, FRD also suffer from poor health, high mortality, and abuse^15^.

Amongst the countries with the largest FRD populations in the world, India stands out as it accounts for an estimated 20 million dog bite cases per year and around 20,000 dog-mediated human rabies deaths per annum^8,16,17^. Apart from being a hotspot of dog-mediated rabies deaths, dog attacks also result in direct human fatalities in India (e.g. https://www.nationalheraldindia.com/india/stray-dogs-terror-in-sitapur-six-children-killed-in-one-week accessed on 19/Jun/2020). There is thus a strong and urgent need to control free-roaming dog populations. In general, efforts to control dog populations in India using a variety of lethal and non-lethal methods have been unsuccessful so far, even though this has been attempted for over 200 years^18^. Even in recent times, lethal methods, such as using strychnine poisoning or culling in gas or electric chambers were implemented haphazardly for decades, but without ancillary measures to restrict access to resources and restricting roaming behaviour, the dog populations rebounded^19,20^. These methods were also criticized for being unnecessarily cruel and were subsequently outlawed^18^.

Since 2001, the only legal method of population control in India, involving capture-neuter-vaccinate-release (Animal Birth Control—ABC) was promulgated^18^. As per the World Organisation for Animal Health (OIE), one of the main objectives of dog population control programmes like ABC is to reduce the abundance of FRD^21^. However, as several reports have shown, these measures were neither fully implemented nor evaluated^22,23^. Indeed, almost all ABC programs have only targeted urban centres e.g.^24^, and not succeeded in substantially reducing dog numbers even with small populations^25^. In the rare cases where ABC has been implemented and monitored continuously for over a decade (e.g. Jodhpur, India), model simulations suggest that the program may result in population reduction of ~ 70% over a 13–18 year period in the best case scenario of ~ 85% population coverage^22^.

Dog population management approaches such as the ABC program which mandates surgical sterilisation, requires considerable financial, infrastructural and personnel support. Operationalising any such program therefore requires careful thought and planning for successful implementation. Often however, there is a lack of understanding of the effort required to significantly and sustainably reduce dog populations. Indeed, the general perception is that a one-time or short burst of surgical interventions will result in permanent eradication or “stray dog free” cities (e.g., https://www.royalpatiala.in/mission-patiala-to-be-first-stray-dog-free-city-bhullar/ accessed on 22/Jun/2020). Government authorities and non-government organizations routinely report the number of surgeries performed as a measure of success, without any mention of the baseline population size, nor track population size subsequent to the ABC campaign. There is thus a strong need for managers to understand the context and set realistic targets, so that the success of the ABC program can be monitored.

Population models that allow for simulation of various scenarios can be used for effective planning and monitoring of dog population management programs. If properly parameterised, such models can be used to understand the scale of effort needed to achieve a set target reduction in population or to understand the challenges that emerge from improperly planned interventions. However, models are rarely, if ever, used by government agencies for scenario building and planning. There are several reasons behind this, including a lack of technical expertise, and the perception that such models are the domain of mathematical experts^26^. Indeed, for the most part, this perception is justified because most existing models are either narrowly parameterised to represent just one geographical area, e.g.^27^, or do not incorporate complexities that represent real-world scenarios e.g.^28–30^. More importantly, they are not sufficiently customisable and portable to simulate multiple scenarios, nor user-friendly for non-modelers to use as a decision support tool.

**Figure 1:**
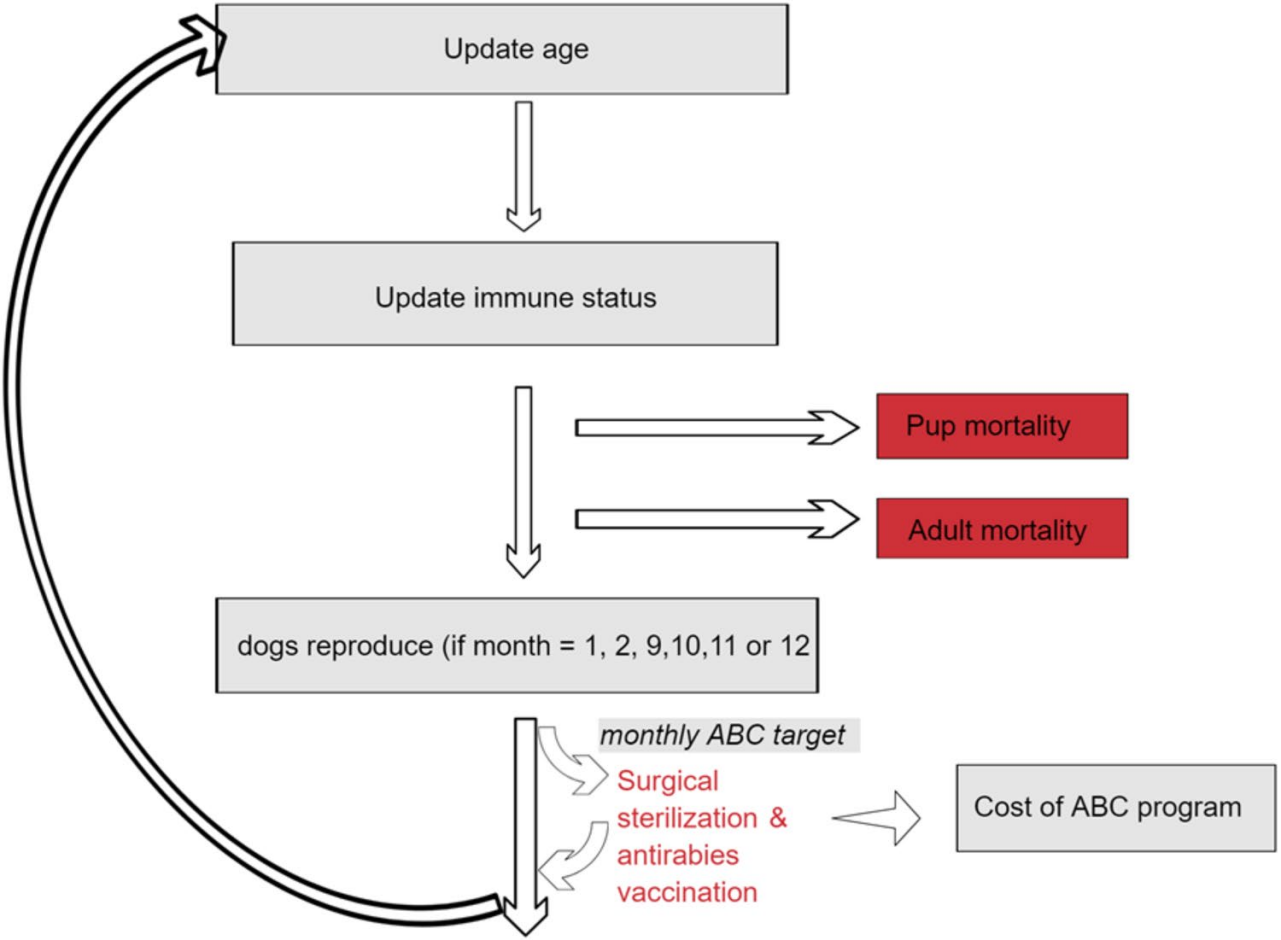
Sequence of events during each step of DogPopDy.

To overcome these limitations, we have developed a novel agent-based modelling tool that generates a realistic in silico dog population, and projects it over a desired number of years. Our user-friendly quantitative evaluation tool allows for locally relevant parameterisation, and simulation of alternate scenarios to help managers understand the possible outcomes of proposed dog population management strategies. Model-generated dog populations incorporate individual attributes and characteristics (like age, sex, reproductive status, accessibility, catchability, age-specific mortality) that underpin heterogeneity observed in the real-world FRD populations. Here we demonstrate the use of this tool to (1) evaluate the success as well as cost-effectiveness of FRD population management interventions like ABC, and (2) to assess if adequate vaccination coverage is achieved for effective rabies control. Specifically, we examine the effect of ABC interventions on FRD abundance and recruitment in the population and population-level anti-rabies coverage (Fig. 1).

## Results

### Model evaluation

**Figure 2:**
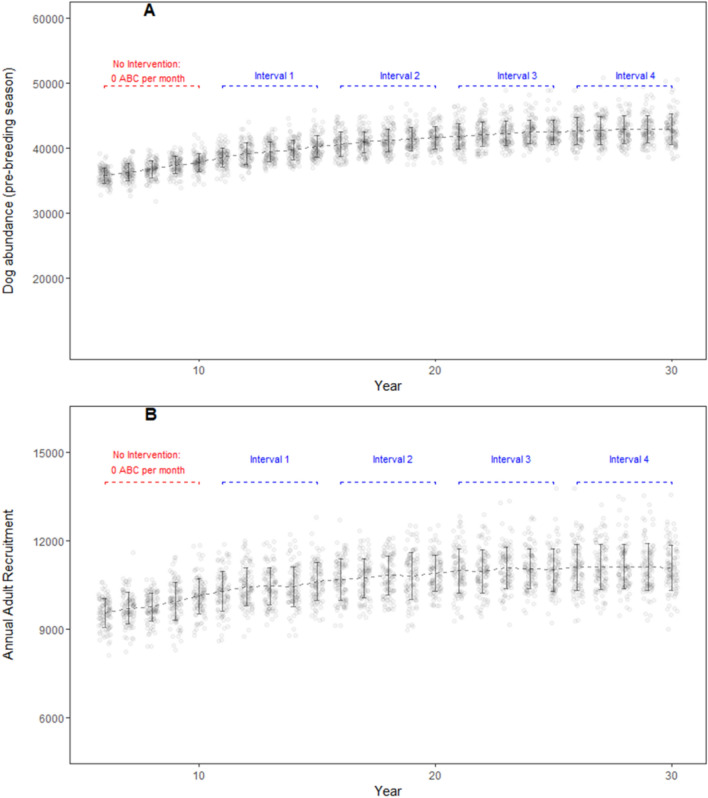
DogPopDy evaluation using a ‘business as usual’ scenario (no population control intervention). Model dog population was projected over a 30 year period. (**A**) Dog abundance (pre-breeding season, month = September), and (**B**) Annual recruitment of juveniles into the adult age class, summarized for 100 DogPopDy iterations. The four assessment intervals are indicated by dashed lines (blue).

Without any population control interventions, the model dog population increased over the course of 25 years from 35,183 (± 1170 SD; year 5) to 42, 879 (± 2318 SD; year 30) (Fig. 2A). Adult dog abundance in the model landscape (enumerated in the first month of each year) reached carrying capacity in year 16 (30,186 ± 1191 SD; 95% CI 29,950–30,423, t(99) = − 0.97901, *p* = 0.33; one-sample *t* test) and remained above the carrying capacity till end of simulation at year 30 (31,956 ± 1570 SD). Annual recruitment in the adult age class increased from 9412 (± 508 SD; year 5) to 11,090 (± 771 SD; year 30) (Fig. 2B).

### Sensitivity analysis

Local sensitivity analysis indicated that dog abundance levels were particularly sensitive to juvenile mortality rate and mean litter size, and to a lesser extent to adult mortality rate (Table 1).

**Table 1:** Sensitivity analysis results for mean pre-breeding season abundance during assessment interval 1 (years 11–15).

Parameter	Range	Assessment interval 1	
		S^+^	S^−^
Adult-mortality-probability	[0.027, 0.029, 0.031]	1.33	− 1.08
Juvenile-mortality-probability	[0.091, 0.101, 0.112]	3.13	− 1.64
Mean litter size	[3.5, 4, 4.5]	− 1.7	2.61
Human:dog ratio	[32, 33, 34]	1.27	− 0.44

DogPopDy was simulated without ABC intervention and 100 model iterations were analyzed to derive the sensitivities.

### Model application

#### Best case scenario—Low intensity ABC effort

**Figure 3:**
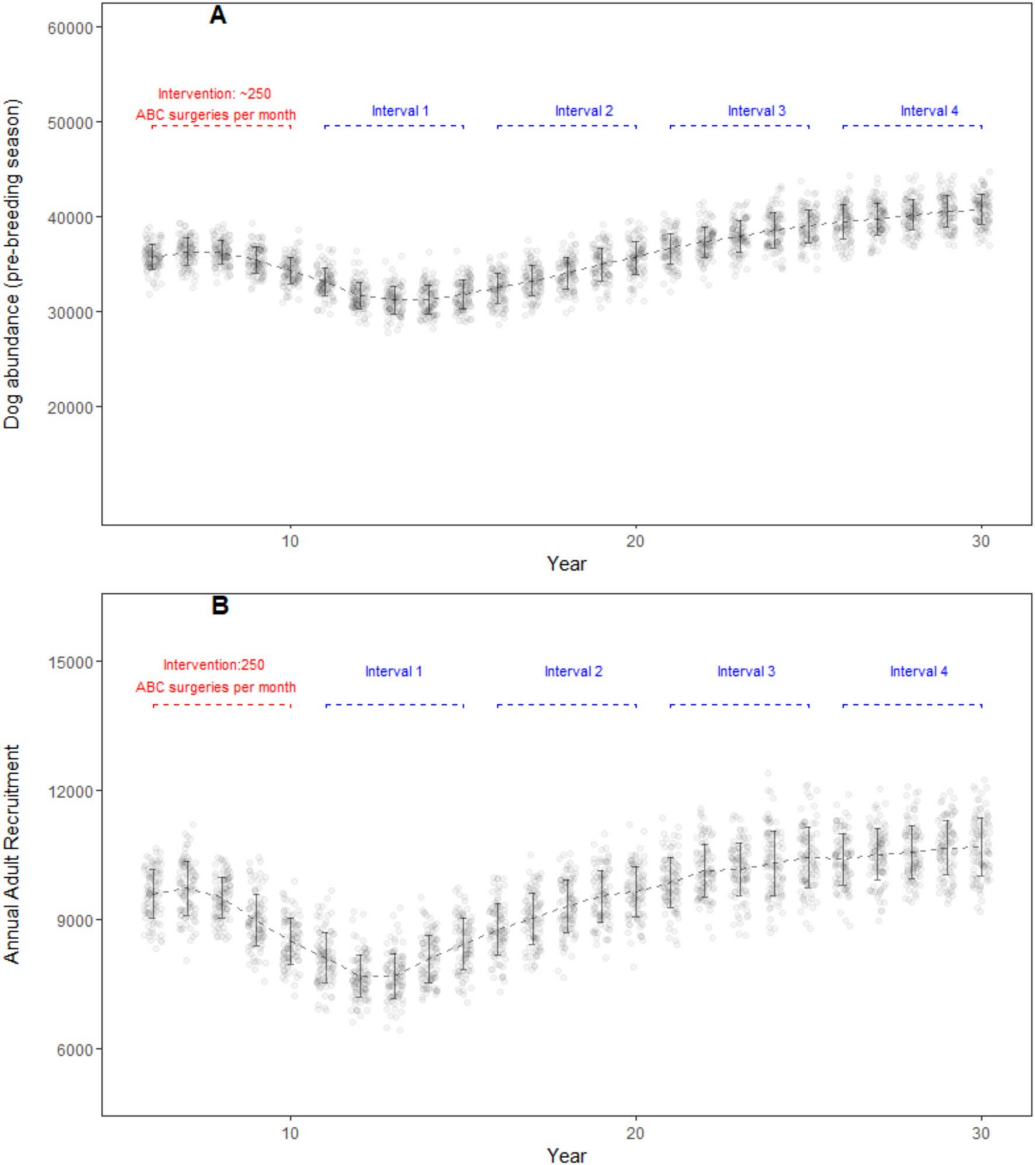
DogPopDy evaluation using a ‘best case’ scenario (closed population, all dogs in the population are equally and easily catchable) with a 5 year ABC program (1 ABC center =  ~ 250 ABC surgeries per month). (**A**) Dog abundance (pre-breeding season, month = September), and (**B**) Annual recruitment of juveniles into the adult age class, summarized for 100 DogPopDy iterations. The four assessment intervals are indicated by dashed lines (blue).

In the ‘best case’ scenario an average of 14,687 ABC surgeries were performed per model iteration, incurring a cost of US$170,426. Model dog population decreased over a 9 year period from 35,121 (± 1,329 SD; year 5) to 31,254 (± 1512 SD; year 14), but by year 20 the dog population was at pre-intervention levels and continued to increase thereafter (40,686 ± 1,614 SD; year 30) (Fig. 3A). The adult proportion of the model dog population also decreased over a 10-year period from 26,656 (± 948 SD; year 6) to 23,830 (± 1,095 SD; year 15), and increased thereafter until it reached carrying capacity by year 29 (30,145 ± 1137 SD; 95% CI 29,919–30,371, t(99) = − 1.389, *p* = 0.168; one-sample *t* test). Annual recruitment in the adult age class decreased from 9,359 (± 634 SD; year 5) to 8,439 (± 589 SD; year 15) over a 10 year period; and increased thereafter over the course of model run (10,689 ± 679 SD; year 30) (Fig. 3B). This best case low-intensity ABC scenario resulted in a maximum anti-rabies vaccination coverage of 8% during the intervention period. The vaccination coverage waned rapidly within a year after the intervention period.

#### Best case scenario—Moderate intensity ABC effort

**Figure 4:**
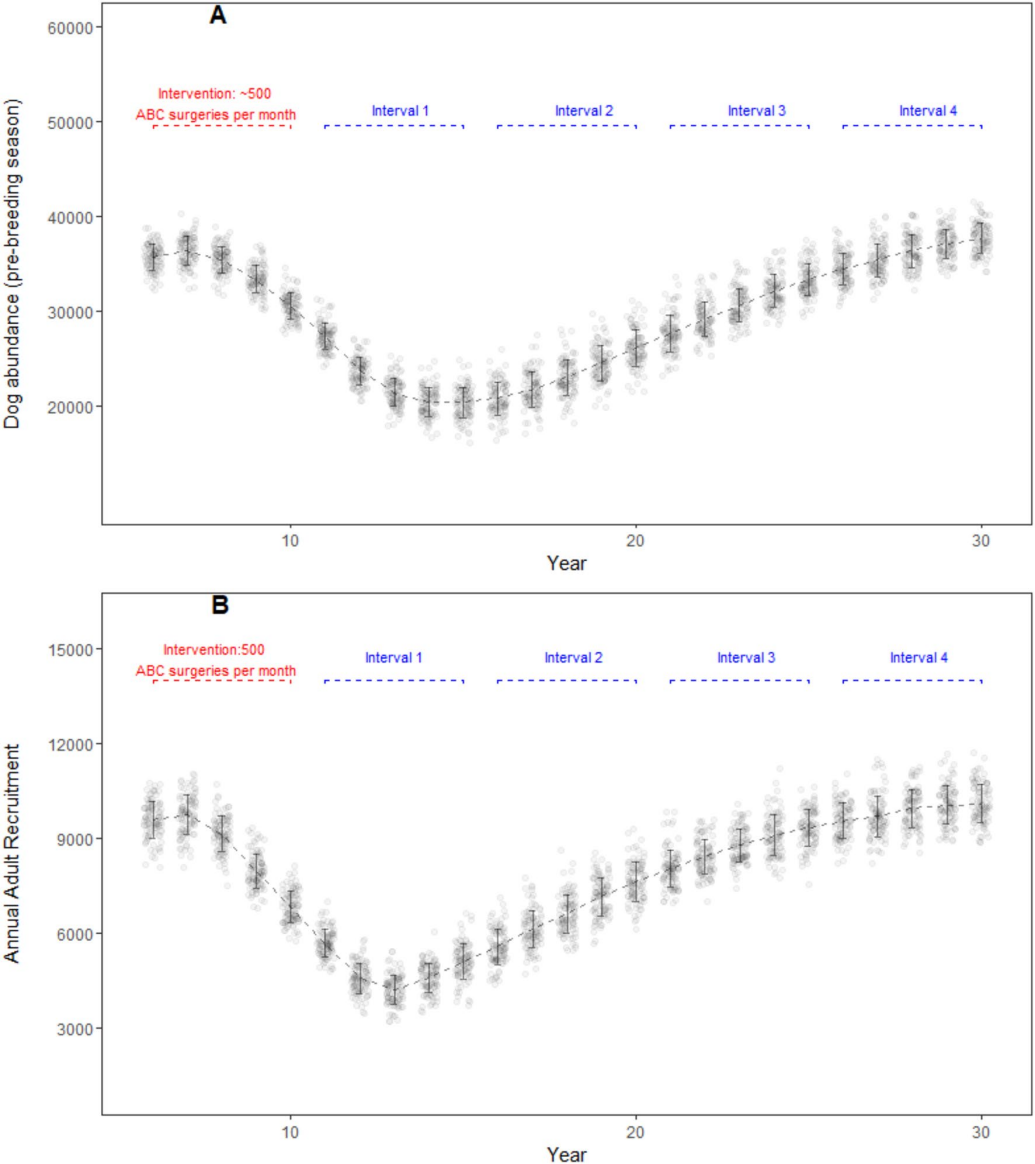
DogPopDy assessment of a moderate intensity ABC effort using a ‘best case’ scenario (closed population, all dogs in the population are equally and easily catchable). Two ABC centers (~ 500 ABC surgeries per month) represented moderate intensity ABC effort. (**A**) Dog abundance (pre-breeding season, month = September), and (**B**) Annual recruitment of juveniles into the adult age class, summarized for 100 DogPopDy iterations. The four assessment intervals are indicated by dashed lines (blue).

An average of 29,380 ABC surgeries per iteration of the moderate intensity 5-year ABC effort incurred a cost of US$340,918. Model dog population decreased from 34,980 (± 1,178 SD; year 5) to 20,306 (± 1,657 SD; year 15) over a 10 year period, but by year 26 the dog abundance had increased to pre-intervention level (34,447 ± 1,660 SD), and continued to increase thereafter (37,650 ± 1,588 SD; year 30) (Fig. 4A). The adult proportion of the model dog population initially decreased over a 10-year period from 26,629 (± 908 SD; year 6) to 15,561 (± 1227 SD; year 15), but increased thereafter until it reached pre-intervention levels by year 27 (26,310 ± 1,219 SD). The adult dog abundance remained below the carrying capacity throughout the model run. Annual recruitment in the adult age class decreased from 9,297 (± 576 SD; year 5) to 5,105 (± 565 SD; 15) over a 10 year period; and increased thereafter over the course of model run (10,111 ± 621 SD; year 30) (Fig. 4B). The maximum anti-rabies vaccination coverage achieved with a moderate-intensity ABC scenario was 18%, but the coverage rapidly waned within a year after the intervention period.

#### Best case scenario—High intensity ABC effort

**Figure 5:**
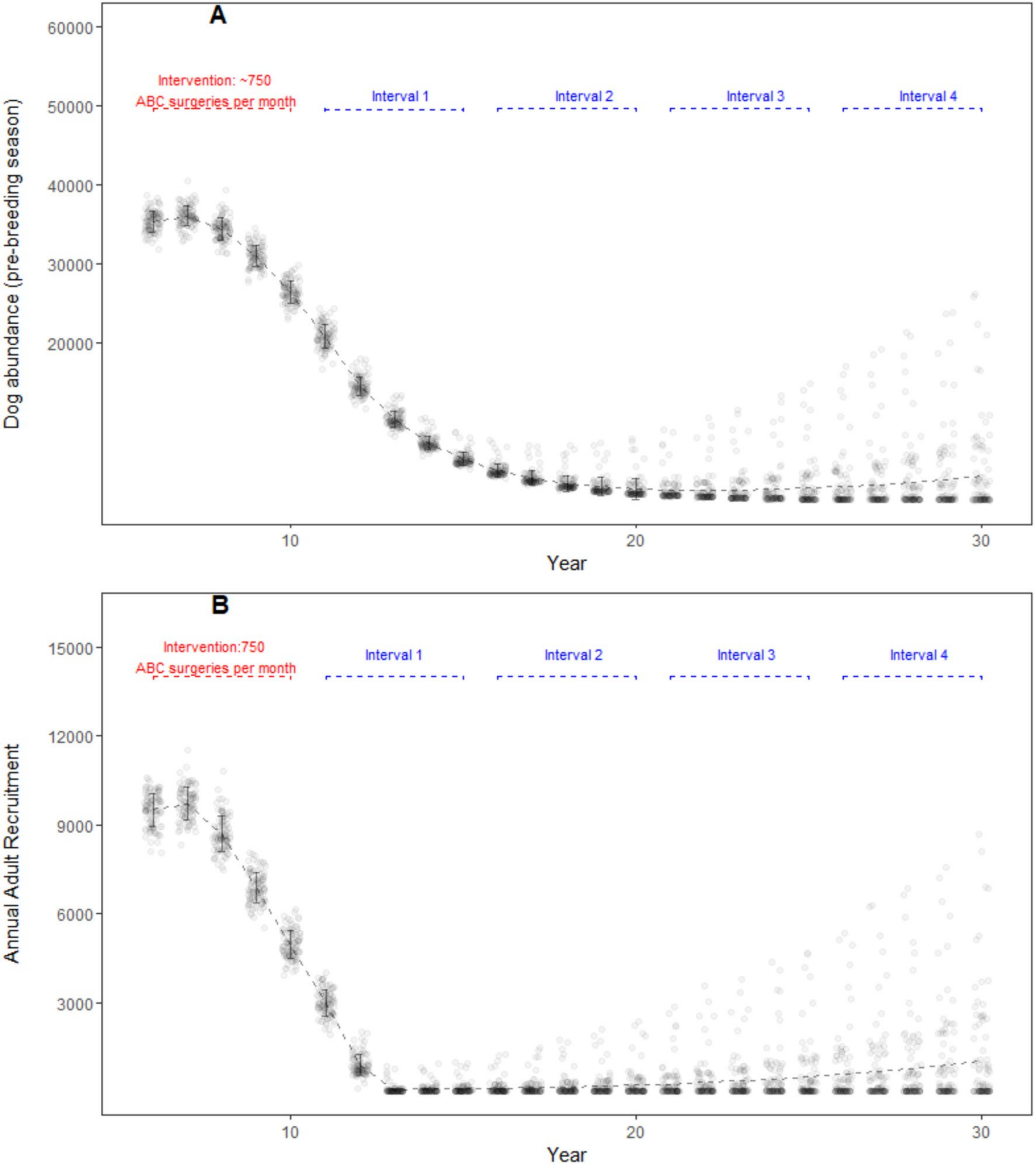
DogPopDy assessment of a high intensity ABC effort using a ‘best case’ scenario (closed population, all dogs in the population are equally and easily catchable). Three ABC centers (~ 750 ABC surgeries per month) represented high intensity ABC effort. (**A**) Dog abundance (pre-breeding season, month = September), and (**B**) Annual recruitment of juveniles into the adult age class, summarized for 100 DogPopDy iterations. The four assessment intervals are indicated by dashed lines (blue).

High intensity ABC effort resulted in an average of 42,608 ABC surgeries per iteration and incurred a cost of US$492,682. Model dog population decreased from pre-intervention abundance of 34,647 (± 1,084 SD; year 5) to 1,447 (± 1,359 SD; year 20), and gradually increased thereafter until year 30 (3,032 ± 5,532 SD; year 30) (Fig. 5A). After the second assessment interval, mean dog abundance values were generally low (< 3000) but overdispersed as indicated by the standard deviations. Nine out of 100 iterations had > 10,000 dogs (with a maximum value of 28,136) in the 30th year. The, adult dog abundance mirrored this decreasing trend (from 26,394 ± 863 SD in year 6 to 1,161 ± 2,140 SD in year 25) but increased to 2,248 ± 4,102 SD by the 30th year. Only six iterations out of 100 had adult dog abundance above 10,000 in the 4th assessment interval. High intensity ABC effort was able to keep the adult dog abundance below the carrying capacity throughout the model run. Annual recruitment in the adult age class decreased from 9,247 (± 540 SD; year 5) to 87 (± 195 SD; year 15) over a 10 year period; and gradually increased thereafter over the course of model run (1,019 ± 1,811 SD; year 30) (Fig. 5B). The maximum anti-rabies vaccination coverage achieved with a high-intensity ABC scenario was 35%, but the coverage rapidly waned within a year after the intervention period.

#### Real world scenario—High intensity ABC effort

**Figure 6:**
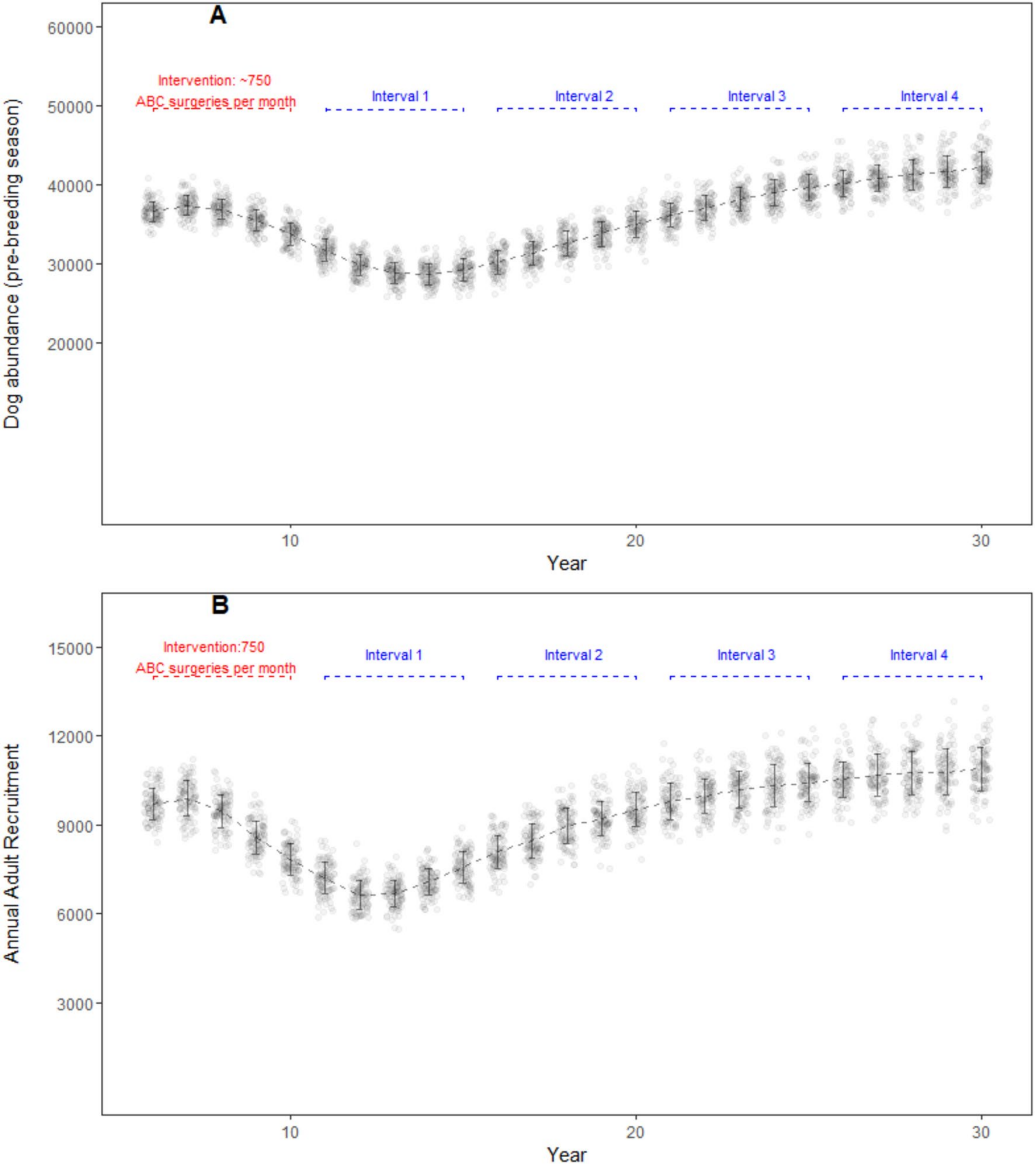
DogPopDy assessment of a high intensity ABC effort using a ‘real world’ scenario (open population, capture heterogeneity, 5% dogs inaccessible for population management interventions). The actual number of ABC surgeries performed in a month is influenced by the catchability of the intact dogs in the population. Furthermore, inaccessible dogs and immigration also influence the population dynamics. (**A**) Dog abundance (pre-breeding season, month = September), and (**B**) Annual recruitment of juveniles into the adult age class, summarized for 100 DogPopDy iterations. The four assessment intervals are indicated by dashed lines (blue).

The high intensity ABC effort was not effective when real world processes were incorporated in the model simulation. An average of 21,099 ABC surgeries per iteration incurred a cost of US$ 243,393. Model dog population decreased from pre-intervention abundance of 35,829 (± 1154 SD; year 5) to 29,150 (± 1439 SD; year 15), increased thereafter surpassing the pre-intervention abundance, and was 42,095 (± 2038 SD) in year 30 (Fig. 6A). The adult dog abundance decreased initially from 27,442 ± 850 SD in year 6 to 21,945 ± 975 SD in year 14, and increased thereafter, eventually surpassing the carrying capacity in year 28 (30,874 ± 1,339 SD) and reached 31,444 ± 1,437 SD in year 30. Annual recruitment in the adult age class decreased from 9,386 (± 556 SD; year 5) to 7,556 (± 526 SD; year 15), then increased over the course of model run (10,878 ± 744 SD; year 30) (Fig. 6B). The maximum anti-rabies vaccination coverage achieved with a high intensity ABC scenario with real world processes was only 9%, and the coverage rapidly waned within a year after the intervention period.

## Discussion

The presence of a large, FRD population has double negative consequences—high public health problems as well as poor animal welfare outcomes. There is thus a strong need for effective management of dog populations in developing countries. According to WHO^31^, the goal of ABC programs is to “reduce dog population turnover as well as the number of dogs susceptible to rabies and limit aspects of male dog behaviour (such as dispersal and fighting) that facilitate the spread of rabies”^31^. Clearly, it would seem that reducing the population of FRD would result in considerable savings as well as a more sustainable program for elimination of rabies^32^. The successful elimination of rabies in many countries in the early 1900s involved not just mass vaccination, but also strong pet ownership laws, and the removal of unowned free-ranging dogs. For example, in Japan, between 1925 and 1928 (both years inclusive), 935,771 dogs were vaccinated, and 856,328 “stray” dogs were captured and removed from the population. This, combined with strict rules regarding vaccination, ownership and regulation of pet dogs ensured a reduction in both free-ranging dogs as well as the elimination of canine rabies^33^. Indeed, in the Osaka prefecture, the first epidemic between 1914 and 1921 was controlled wholly through the leashing of pet dogs and the removal of unowned dogs^33^.

Lethal methods for population control have rarely been successful, mainly due to societal barriers, as well as ineffective and incomplete implementation^26^. The use of alternate strategies, such as fertility control, are instead seen as more “humane” and socially acceptable^34,35^. However, in the absence of a systematic planning and monitoring protocol, well intentioned, but poorly planned programs may likely do little good. Our population modelling tool highlights the importance of understanding the dynamics of dog populations, the effort needed to achieve a particular target goal, and the associated costs over a long-period of time. Our simulations show that, even under a limited set of ideal conditions, the target of “zero” reproducing dogs is not achievable within a reasonable timeframe, given the current methods, and more importantly, this method will fail to achieve the goal of eliminating dog-mediated rabies (ZeroBy2030). In our simulations, only the high intensity ABC best case scenario achieved the stated goal of substantially reducing the number of adult dogs in the population (Table 2). However, this required substantial investment of close to half a million US dollars and more than 42,600 surgeries (for an initial dog population of only ~ 34,650) over the 5 year intervention period. Despite such a high rate of intervention, the maximum vaccination coverage achieved did not exceed 35%, which is half the recommended rate by WHO.

**Table 2:** Model dog population parameters (pre-breeding season abundance and annual recruitment in the adult class) compared for five ABC scenarios.

ABC target (per month)	Scenario	Model population parameter (± SD)	Pre-ABC	Year 10	Year 15	Year 20	Year 25	Year 30
No ABC	Business as usual	Abundance	35,183 (± 1,170)	*37,756 (*± *1343)*	*40,178 (*± *1,661)*	*41,573 (*± *1,766)*	*42,420 (*± *1,843)*	*42,879 (*± *2,318)*
		Annual adult recruitment	9,412 (± 508)	*10,131 (*± *593)*	*10,622 (*± *648)*	*10,913 (*± *616)*	*11,011 (*± *710)*	*11,090 (*± *771)*
~ 250	Best case	Abundance	35,121 (± 1,329)	34,271 (± 1,395)	31,781 (± 1,529)	*35,584 (*± *1,785)*	*38,944 (*± *1,762)*	*40,686 (1,614)*
		Annual adult recruitment	9,539 (± 634)	8,512 (± 531)	8439 (± 589)	*9,647 (*± *592)*	*10,441 (705)*	*10,689 (*± *679)*
~ 500	Best case	Abundance	34,980 (± 1,178)	30,507 (± 1,366)	20,306 (± 1,657)	26,069 (± 1,934)	33,265 (± 1,650)	*37,650 (*± *1,588)*
		Annual adult recruitment	9,297 (± 576)	6,831 (± 501)	5,105 (± 565)	7,634 (± 620)	9,244 (± 572)	*10,111 (*± *621)*
~ 750	Best case	Abundance	34,647 (± 1,084)	26,333 (± 1,381)	5,217 (± 833)	1,447 (± 1,359)	1,540 (± 2,886)	3,032 (± 5,532)
		Annual adult recruitment	9,247 (± 540)	4,952 (± 476)	87 (± 195)	211 (± 462)	481 (± 968)	1,019 (± 1,811)
~ 750	Real world	Abundance	35,829 (± 1,154)	33,726 (± 1,416)	29,150 (± 1,439)	34,976 (± 1,608)	*39,577 (*± *1,636)*	*42,095 (*± *2,038)*
		Annual adult recruitment	9,386 (± 556)	7,835 (± 522)	7,556 (± 526)	*9,514 (*± *580)*	*10,422 (*± *639)*	*10,878 (*± *744)*

Business as usual scenario is simulated in a closed dog population without any ABC intervention. For all other scenarios, ABC program is implemented over a 5-year period (year 6 to year 10). Best case scenario assumes a closed population and homogeneous capture probability. Real world scenario simulates 1% net annual immigration into the model dog population and heterogenous capture probability. Italicised values indicate values exceeding pre-intervention level.

We further showed that even if there was a small proportion of the population being inaccessible for capture (5% of the initial adult carrying capacity) and very low levels of immigration (1% net immigration per annum), population control was not achieved, even with very high effort. The peak anti-rabies vaccination coverage remained below 10% even though the cost associated with this program was close to a quarter of a million US dollars. It is important to point out here that the scenarios mentioned above are highly conservative, and that in actuality both the proportion of inaccessible dogs, as well as immigration rates are likely to be much higher. This is because, unlike the situation in countries from the African continent (and other parts of the world), where most dogs are either wholly or partially owned by individuals or communities^36^, in India, a significant proportion of dogs may be unowned (between 18 and 42%)^37–39^. Interventions like ABC or mass vaccination require capture and handling of such dogs. Some dogs are readily accessible, but some require considerably greater effort than regular central point mass vaccination campaigns^39,40^. An assumption of equal catchability implies that monthly ABC targets from a particular region will be readily met. However, if a certain proportion of dogs require additional efforts for catching, then, given the human resources and time available, it is likely to result in shortfalls in the number of dogs captured and sterilised. The infusion of even highly conservative “real world” parameters in our models, renders the ABC exercise futile, both in terms of reducing population size, as well as in achieving sufficient anti-rabies vaccination coverage levels.

The parameters, and results from our model simulations, are not far from reality. For example, the amount of financial resources allocated by cities is typically half of what was required in our “real-world” scenario. The city of Nashik in western India, allocated a budget of ~ US$ 141,000 (1 crore INR, https://timesofindia.indiatimes.com/city/nashik/civic-body-finalizes-agency-for-stray-dog-sterilization/articleshow/71086742.cms accessed on 22/Jun/2020) for a city with a human population of 1.5 million, and a derived dog population of ~ 45,000 (based on 33:1 human:dog ratio). There was no mention of estimating the actual population size, which is a common lacuna in most such exercises^41^, and thus no target reduction in dog population size. The assumption from the city planners is that this operation will yield results similar to that achieved by the “high intensity best-case scenario” (Fig. 5), but given the realities on the ground, the result is more likely to be similar to Fig. 6. Furthermore, breaks in or discontinuation of, the ABC program can result in even lower coverage levels and reduce the effectivity of the program (e.g. https://timesofindia.indiatimes.com/city/guwahati/birth-control-scheme-for-stray-dogs-hits-roadblock-due-to-limited-funds/articleshow/69081059.cms accessed on 22/Jun/2020). Thus, a lot of effort, resources and time will have been spent, without any significant impact in either reducing dog populations or in achieving sufficient vaccination coverage.

Efforts to combat rabies mainly focus on mass dog vaccination campaigns^42,43^. The World Health Organisation and other leading international agencies have advocated annually vaccinating 70% of the dog population to break transmission cycles. With this strategy, the WHO and other organisations aim to eliminate dog-mediated human rabies deaths by 2030^44^. Several pilot and scale projects have shown promising results using this strategy, giving hope that achieving “Zero by 2030” may not be such an ambitious target^45^. However, as we have shown, if this strategy is combined with an ABC program to simultaneously reduce dog populations, it is logistically unfeasible to achieve the necessary coverage levels.

Mass vaccination without ABC is advocated as an alternate mechanism to achieve 70% coverage levels^46,47^. Anti-rabies vaccination has been shown to also reduce all-cause mortality in a cohort of dogs in South Africa^48^. The sensitivity analysis on our model shows that dog population is sensitive to both litter size and juvenile mortality. Thus, it is likely that in the absence of an effective population control measure, only vaccination campaigns may end up in increasing dog populations. This is problematic for several reasons, including, in hampering the success of future anti-rabies vaccination programs. At current levels of vaccine production and the financial resources committed, Wallace et al.^49^ estimate a vaccine shortfall of 7.5 billion doses and a resource gap of US$ 3.9 billion to achieve global dog rabies elimination by 2030^49^. This analysis also does not take in to account that a substantial proportion of unowned dogs may fail to sero-convert to the required protective antibody levels with a single dose of vaccine^50^. It therefore becomes imperative, in the fight against rabies, to also not lose focus on effective and long-term solutions to dog population management.

Our model simulation tool allows for relatively easy manipulation of all key parameter settings to test for capacity to effectively reduce populations within a reasonable period of time. The tool, developed as a customizable agent-based model (DogPopDy), can incorporate real-world processes like density-dependent survival, capture heterogeneity and immigration. The model program has a user-friendly Graphical User Interface (GUI), and the interface sliders and choices allow users (even non-modelers) to update model assumptions and perform virtual experiments. Practitioners and civic agencies can employ model-based explorations to estimate the amount of effort needed to achieve a particular target objective, as well as the time and effort needed to maintain the population at the target levels. It allows public authorities to incorporate defensible decisions while planning and implementing dog population management programs. Potentially the tool can also be modified and developed to model the impact of vaccination strategies under different population density scenarios, as well serve as a decision support system for strategic intervention planning. Here, we have simulated relatively simple scenarios, with highly conservative parameter estimates. However, our model tool allows for more complex scenario building with better estimates of population vital rates, breaks in ABC programs (due to funding cuts or other reasons), spatial heterogeneity in ABC programs, implementation of responsible dog ownership (through a removal option) or in planning long-term programs with set population targets, re-vaccination strategies, and the reduction of carrying capacity. The use of this tool thus has the potential to bring a much needed dose of realism in understanding the constraints and challenges in managing free-ranging dog populations in the real world.

## Methods

### Model description

The model, DogPopDy, was developed in NetLogo 6.0.4^51^. NetLogo is a software platform for implementing agent-based models. Model description is provided following the Overview, Design concepts, Details (ODD) protocol for individual-based models^52,53^. Model code has been peer-reviewed and published as open access and is available via website repository “Open ABM CoMSES Computational Model Library” (https://doi.org/10.25937/9nge-4s45). The complete ODD Protocol is provided in Supplementary Materials 1. The model components and sequence of events are shown in Fig. 1.

We initialized DogPopDy with a human population of 1,000,000 and a human:dog ratio of 33:1 to represent a typical urban area in India. The initial number of adult dogs in the model landscape is determined by dividing the human population by the human–dog ratio. This is referred to as the adult dog carrying capacity for the model landscape. Adult dog carrying capacity is not explicitly enforced anytime during the model run but is used to simulate density-dependent juvenile mortality (See ODD protocol 1.6.6). Age is set at 13 months or older for all dogs in the model during the setup. We run the model for 5 years (“burn-in” period) to achieve a stable age distribution before simulating ABC interventions and recording model outputs. The model dog population is projected over a 30-year period. To account for stochasticity in the model runs, we undertook 100 iterations for each scenario (described below).

Three model-derived metrics were used to assess the impact of ABC interventions (or lack thereof) on the model dog population: (a) dog abundance (reflecting the pre-breeding season population in the month of September), (b) annual recruitment into the adult class, and (c) anti-rabies vaccination coverage. Specifically, we compared population metrics before and after the intervention period (year 5/6 and years 10, 15, 20, 25, 30).

### Model evaluation and sensitivity analysis

Model performance was first evaluated by simulating a ‘*business as usual*’ scenario (no ABC program) in a closed dog population (no immigration or emigration). There was no anti-rabies vaccination coverage in the ‘*business as usual*’ scenario.

We then performed a local sensitivity analysis of dog abundance (mean pre-breeding season abundance) during assessment interval 1 (years 11 to 15). Sensitivity values were generated for select parameters following steps outlined in Railsback and Grimm^54^. We examined sensitivity of the model outcomes to the parameters related to mortality rates (adult mortality and juvenile mortality), reproduction (mean litter size), and carrying capacity (human:dog ratio). A range was constructed for each parameter analyzed such that the lower (R^−^) and upper (R^+^) endpoints were within 5–15% of the reference value (R). One hundred iterations of DogPopDy without ABC were undertaken for each parameter value to generate sensitivity values. The sensitivities were calculated as follows:

$$\begin{aligned} {\text{S}}^{ + } & = {{\left( {\left( {{\text{C}}^{ + } - C} \right)/C} \right)} \mathord{\left/ {\vphantom {{\left( {\left( {{\text{C}}^{ + } - {\text{C}}} \right)/{\text{C}}} \right)} {\left( {\left( {{\text{R}}^{ + } - {\text{R}}} \right)/{\text{R}}} \right)}}} \right. \kern-\nulldelimiterspace} {\left( {\left( {{\text{R}}^{ + } - {\text{R}}} \right)/{\text{R}}} \right)}} \\ {\text{S}}^{ - } & = {{\left( {\left( {{\text{C}}^{ - } - {\text{C}}} \right) \, /{\text{C}}} \right)} \mathord{\left/ {\vphantom {{\left( {\left( {{\text{C}}^{ - } - {\text{C}}} \right) \, /{\text{C}}} \right)} {\left( {\left( {{\text{R}}{-}{\text{R}}^{ - } } \right)/{\text{R}}} \right)}}} \right. \kern-\nulldelimiterspace} {\left( {\left( {{\text{R}}{-}{\text{R}}^{ - } } \right)/{\text{R}}} \right)}} \\ \end{aligned}$$

where C^−^, C and C^+^ are average dog abundance values when a parameter is valued at R^−^, R and R^+^, respectively.

### Model application

We used DogPopDy to assess the impact of ABC effort intensity (number of ABC surgeries per month) on the efficacy of dog population management programs. We implemented a 5-year ABC program with 1 ABC center (~ 250 ABC surgeries per month) in the closed model population where all dogs were equally and easily catchable (‘best case’ scenario). We also simulated two additional levels of ABC efforts: moderate intensity (2 ABC centers ~ 500 ABC surgeries per month for 5 years) and high intensity (3 ABC centers ~ 750 ABC surgeries per month for 5 years). We further evaluated high intensity of ABC effort in a real-world context. Specifically, we incorporated capture heterogeneity (see *dog sterilization*) in the model simulation, designated 5% dogs in the model population as inaccessible for ABC intervention, and set the net annual immigration of dogs into the model population at 1%. The results of all scenarios are summarised in Table 2.

For the best case scenario (closed population, all dogs are equally and easily catchable), stochasticity in the dog capturing process is modelled by decreasing the monthly target by up to 5%. We assume that the monthly target so derived is equally divided between males and females.

For the real-world scenario, we incorporate processes that affect dog captures, and therefore the actual number of ABC surgeries per month. Capture effort heterogeneity is an important factor influencing the number of dogs captured for the ABC program. While planning dog population management strategies, it is often assumed that all dogs are catchable. However, in the real world, the capture effort, and therefore the capture efficiency, varies between dogs. Some dogs are easy to capture (cooperative reference persons, friendly community-owned dogs accustomed to handling), while others require additional effort and time (capture using nets, cages, chemical immobilization). Based on FRD capture data from rural as well as urban sites^39^ (Vanak unpublished data), we estimated that 40% dogs in free-ranging dog populations are easy to capture while 60% require additional effort than what is normally deployed by the dog catching team. To incorporate capture effort heterogeneity in the model, we have included a dog state variable ‘catchability’. A non-zero catchability between 1 and 100 is randomly assigned to each dog in the model population. Dogs with catchability > 60 have a capture probability of 1 (readily accessible with standard catch effort), dogs with catchability between 30 and 60 have a capture probability of 0.67 (require 50% more effort than standard catch effort), and dogs with catchability below 30 have a capture probability of 0.5 (require 100% more effort than standard catch effort). The proportion of unneutered dogs that are catchable during a time step is determined as follows:

Proportion of unneutered catchable dogs = {(Number of unneutered dogs with catchability > 60 * 1) + (Number of unneutered dogs with catchability between 30 and 60 * 0.67) + (Number of unneutered dogs with catchability < 30 * 0.5)}/Total dogs.

The monthly ABC target is scaled using the proportion of unneutered catchable dogs.

For the real world scenario, it is also possible to designate a proportion of the dog population as inaccessible (using the slider ‘*piad*’). Owned, free-ranging dogs protected by their owners from the dog catching team or truly feral dogs fall in this category. Inaccessible dogs remain intact and unvaccinated throughout the simulation.

The desired target for the ABC program can be set using slider ‘*target-reduction*’ (the desired reduction expressed as percent of the initial population). A post-target ABC rate can also be specified (set using slider: *followup-abc-rate*)—this rate is implemented if the desired reduction in dog population is achieved before the ABC program duration is completed. All sterilized dogs are vaccinated against rabies, and the duration of immunity is set at 12 months.

## Supplementary information

Supplementary Information.

## References

[1] Gompper ME, Gompper ME (2014). The dog-human-wildlife interface: assessing the scope of the problem. Free-Ranging Dogs and Wildlife Conservation.

[2] Hughes J, Macdonald DW (2013). A review of the interactions between free-roaming domestic dogs and wildlife. Biol. Conserv..

[3] Jaleta TG (2017). Different but overlapping populations of *Strongyloides stercoralis* in dogs and humans—dogs as a possible source for zoonotic strongyloidiasis. PLoS Negl. Trop. Dis..

[4] Deplazes P, van Knapen F, Schweiger A, Overgaauw PAM (2011). Role of pet dogs and cats in the transmission of helminthic zoonoses in Europe, with a focus on echinococcosis and toxocarosis. Vet. Parasitol..

[5] Ashford DA (1998). Studies on control of visceral leishmaniasis: Impact of dog control on canine and human visceral leishmaniasis in Jacobina, Bahia, Brazil. Am. J. Trop. Med. Hyg..

[6] Quinnell R, Courtenay O (2009). Transmission, reservoir hosts and control of zoonotic visceral leishmaniasis. Parasitology.

[7] Carmena D, Guillermo C (2013). Canine echinococcosis: global epidemiology and genotypic diversity. Acta Trop..

[8] Hampson K (2015). Estimating the global burden of endemic canine rabies. PLoS Negl. Trop. Dis..

[9] Home, C. *et al.* Commensal in conflict: livestock depredation patterns by free-ranging domestic dogs in Upper Spiti Landscape, Himachal Pradesh, India. *Ambio* 1–12 (2017). 10.1007/s13280-016-0858-6 PMC5595737 28074403

[10] Montecino-Latorre D, San Martín W (2019). Evidence supporting that human-subsidized free-ranging dogs are the main cause of animal losses in small-scale farms in Chile. Ambio.

[11] Gompper ME (2014). Free-Ranging Dogs and Wildlife Conservation.

[12] Vanak AT, Gompper ME (2009). Dogs as carnivores: their role and function in intraguild competition. Mamm. Rev..

[13] Doherty TS (2017). The global impacts of domestic dogs on threatened vertebrates. Biol. Conserv..

[14] Belsare AV, Vanak AT, Gompper ME (2014). Epidemiology of viral pathogens of free-ranging dogs and Indian foxes in a human-dominated landscape in central India. Transbound. Emerg. Dis..

[15] Jackman, J. & Rowan, A. Free-roaming dogs in developing countries: The benefit of capture, neuter and return programs. *State Anim. IV* 55–78 (2007).

[16] Sudarshan MK (2007). Assessing the burden of human rabies in India: results of a national multi-center epidemiological survey. Int. J. Infect. Dis..

[17] Gongal G, Wright AE (2011). Human rabies in the WHO Southeast Asia region: forward steps for elimination. Adv. Prev. Med..

[18] Nadal D, Lynteris C (2019). To kill or not to kill? Negotiating life, death, and One Health in the context of dog-mediated rabies control in colonial and independent India. Framing Animals as Epidemic Villains.

[19] AWBI. *Revised module for street dog population management, rabies eradication, reducing man-dog conflict* (2016). https://awbi.org/awbi-pdf/revised_abc_module.pdf.

[20] Morters MK (2013). Evidence-based control of canine rabies: a critical review of population density reduction. J. Anim. Ecol..

[21] OIE. *Stray dog population control*. *Terrestrial Animal Health Code* (2015).

[22] Totton SC (2010). Stray dog population demographics in Jodhpur, India following a population control/rabies vaccination program. Prev. Vet. Med..

[23] Uniyal, M. & Vanak, A. T. Barking up the wrong tree: the agency in charge of controlling street dogs is completely ineffective. *Scroll* (2016).

[24] Hiby LR (2011). A mark-resight survey method to estimate the roaming dog population in three cities in Rajasthan, India. BMC Vet. Res..

[25] Belo VS (2017). Abundance, survival, recruitment and effectiveness of sterilization of free-roaming dogs: a capture and recapture study in Brazil. PLoS ONE.

[26] Smith LM (2019). The effectiveness of dog population management: a systematic review. Animals.

[27] Yoak AJ, Reece JF, Gehrt SD, Hamilton IM (2016). Optimizing free-roaming dog control programs using agent-based models. Ecol. Model..

[28] Garde E, Pérez GE, Vanderstichel R, Dalla Villa PF, Serpell JA (2016). Effects of surgical and chemical sterilization on the behavior of free-roaming male dogs in Puerto Natales, Chile. Prev. Vet. Med..

[29] Kisiel LM (2018). Modeling the effect of surgical sterilization on owned dog population size in Villa de Tezontepec, Hidalgo, Mexico, using an individual-based computer simulation model. PLoS ONE.

[30] Santos Baquero O, Akamine LA, Amaku M, Ferreira F (2016). Defining priorities for dog population management through mathematical modeling. Prev. Vet. Med..

[31] WHO. *WHO Expert Consultation on Rabies: first report*. *World Health Organization Technical report series* vol. 931 (2004). 16485446

[32] Collinson A, Bennett M, Brennan ML, Dean RS, Stavisky J (2020). Evaluating the role of surgical sterilisation in canine rabies control: a systematic review of impact and outcomes. PLoS Negl. Trop. Dis..

[33] Kurosawa A (2017). The rise and fall of rabies in Japan: a quantitative history of rabies epidemics in Osaka Prefecture, 1914–1933. PLoS Negl. Trop. Dis..

[34] Yoak AJ, Reece JF, Gehrt SD, Hamilton IM (2014). Disease control through fertility control: secondary benefits of animal birth control in Indian street dogs. Prev. Vet. Med..

[35] Kartal T, Rowan AN, Polak K, Kommedal AT (2018). Stray dog population management. Field Manual for Small Animal Medicine.

[36] Lembo T (2010). The feasibility of canine rabies elimination in Africa: dispelling doubts with data. PLoS Negl. Trop. Dis..

[37] Byrnes H, Britton A, Bhutia T (2017). Eliminating dog-mediated rabies in Sikkim, India: a 10-year pathway to success for the SARAH program. Front. Vet. Sci..

[38] Fitzpatrick MC (2016). One Health approach to cost-effective rabies control in India. Proc. Natl. Acad. Sci. U. S. A..

[39] Belsare AV, Gompper ME (2013). Assessing demographic and epidemiologic parameters of rural dog populations in India during mass vaccination campaigns. Prev. Vet. Med..

[40] Gibson AD (2020). Reviewing solutions of scale for canine rabies elimination in India. Trop. Med. Infect. Dis..

[41] Tiwari HK, Vanak AT, O’Dea M, Gogoi-Tiwari J, Robertson ID (2018). A comparative study of enumeration techniques for free-roaming dogs in rural Baramati, District Pune, India. Front. Vet. Sci..

[42] Cleaveland S, Hampson K, Lembo T, Townsend S, Lankester F (2014). Role of dog sterilisation and vaccination in rabies control programmes. Vet. Rec..

[43] Lankester F (2014). Implementing pasteurs vision for rabies elimination. Science.

[44] Abela-Ridder B (2016). 2016: the beginning of the end of rabies?. Lancet Glob. Health.

[45] Cleaveland S, Hampson K (2017). Rabies elimination research: Juxtaposing optimism, pragmatism and realism. Proc. R. Soc. B Biol. Sci..

[46] Gibson AD (2015). Vaccinate-assess-move method of mass canine rabies vaccination utilising mobile technology data collection in Ranchi, India. BMC Infect. Dis..

[47] Gibson AD (2018). One million dog vaccinations recorded on mHealth innovation used to direct teams in numerous rabies control campaigns. PLoS ONE.

[48] Knobel DL (2017). Rabies vaccine is associated with decreased all-cause mortality in dogs. Vaccine.

[49] Wallace RM, Undurraga EA, Blanton JD, Cleaton J, Franka R (2017). Elimination of dog-mediated human rabies deaths by 2030: needs assessment and alternatives for progress based on dog vaccination. Front. Vet. Sci..

[50] Pimburage RMS, Gunatilake M, Wimalaratne O, Balasuriya A, Perera KADN (2017). Sero-prevalence of virus neutralizing antibodies for rabies in different groups of dogs following vaccination. BMC Vet. Res..

[51] Wilensky U, Evanston I (1999). NetLogo: Center for Connected Learning and Computer-Based Modeling.

[52] Grimm V (2006). A standard protocol for describing individual-based and agent-based models. Ecol. Model..

[53] Grimm V (2010). The ODD protocol: a review and first update. Ecol. Model..

[54] Railsback SF, Grimm V (2011). Agent-Based and Individual-Based Modeling: A Practical Introduction.

