# Supplementary material for: Modelling the challenges of managing free-ranging dog populations
Source: Sci Rep. 2020 Nov 2;10:18874. doi: 10.1038/s41598-020-75828-6 (PMC7608676; doi:10.1038/s41598-020-75828-6)
Supplement: Supplementary file 1 — Supplementary Information. [file 41598_2020_75828_MOESM1_ESM.pdf]

Modelling the challenges of managing free-ranging dog populations

Aniruddha Belsare, Boone & Crockett Quantitative Wildlife Center, Department of Fisheries & Wildlife, Michigan State University, East Lansing, Michigan, USA.

OneHealth Working Group, Center for Modeling Complex Interactions, University of Idaho, Moscow, Idaho, USA.

belsare1@msu.edu

Abi Tamim Vanak, Ashoka Trust for Research in Ecology and the Environment, Bangalore, India.

DBT/ Wellcome Trust India Alliance Program (Clinical and Public Health Fellowship), Hyderabad, India

School of Life Sciences, University of KwaZulu-Natal, Durban, South Africa.

*avanak@atree.org

Supplementary Information 1.

ODD Protocol

1 Model description

The model, DogPopDy, was developed in NetLogo 6.0.4 [1]. NetLogo is a software platform for implementing agent-based models. Model description is provided following the ODD (Overview, Design concepts, Details) protocol for individual-based models [2,3]. Model code is available via website repository “Open ABM CoMSES Computational Model Library” (https://www.comses.net/codebase-release/dd1d9996-0042-4aaf-b645-8723307fe61c/).

1.1 Purpose

The purpose of this model is to create an in silico FRD population resembling a typical population in urban/rural areas of any country to design and evaluate dog population management and rabies control strategies.

1.2 Entities, State Variables and Scales

Entities: The model has two entities: dogs and patches. Dogs are modeled as individuals occurring on the patches in the model landscape. State variables for the two entities are described in Suppl. **Table 1**.

Spatial scale: The model landscape represents a 400 square kilometer area (20 x 20 patches, each patch equals 1 square kilometer).

Temporal scale: The model has a monthly time step and is simulated for 30 years.

1.3 Process overview and scheduling

The model simulates demographic processes (birth, aging, migration and death) and ABC intervention (surgery+rabies immunisation) in the model dog population (**Figure 1**). During each time step, the age and anti-rabies immunity status of agents (dogs) is updated. The age-specific probability of death during each time step is derived from annual mortality rates (See Submodels section for details).

Reproduction is seasonal; female dogs older than 8 months of age breed between September (month 9) and February (month 2). The sterilization submodel (ABC program) is implemented after the model runs for five years (burn-in period). A proportion of accessible, intact dogs are neutered and vaccinated against rabies every month for the duration of the ABC program.

1.4 Design Concepts

Emergence: The emergent effect of interest here is the population size as a consequence of population management (ABC) efforts in an open dog population with heterogeneous accessibility (catchability). ABC effort is limited by the proportion of inaccessible dogs and immigration rate.

Stochasticity: During each time-step, individuals are selected stochastically to implement three processes: reproduction, mortality and sterilization. Number of pups in each litter is determined by randomly selecting a value between one and six.

Observation: The following observations are updated and documented during each time-step: dog population abundance (graph and monitor; Fig. S1), number of ABC surgeries per month (graph), proportion of neutered individuals in the population (monitor), cumulative number of ABC surgeries (monitor) and cumulative percent reduction compared to population size before ABC was initiated (monitor; Fig. S1). Total cost for the entire duration of ABC program (cost per surgery * inflation *

number of surgeries every year) is also reported (monitor). Results of each model run are also written as a .csv file.

1.5 Initialization

The model dog population is created using two parameters, human population (set using slider: *human-population*) and human-dog ratio (set using slider: *human-dog-ratio;* Fig. S1). Human population is divided by the human-dog ratio to determine initial number of adult dogs in the model landscape. This is referred to as the adult dog carrying capacity for the model landscape. Adult dog carrying capacity is not explicitly enforced anytime during the model run but is used to simulate density-dependent juvenile mortality.

Mean dog density (or mean number of dogs per patch) is calculated as follows:

$$Mean\ dog\ density = \frac{Initial\ number\ of\ adult\ dogs}{number\ of\ patches\ in\ the\ model\ landscape}$$

Initial number of dogs on a patch is derived from a normal distribution around mean dog density ± 20% SD.

Age is set at 13 months or older for all dogs in the model during the setup. We therefore run the model for 5 years ("burn-in" period) before simulating ABC interventions and recording model outputs.

The cost per ABC surgery was set at a conservative INR 700 (US$ 10.5) (https://timesofindia.indiatimes.com/city/nashik/civic-body-finalizes-agency-for-stray-dog-sterilization/articleshow/71086742.cms accessed on 20/Jun/2020) with an annual inflation of 5%. However, there is an option in the model for the user to specify initial cost per ABC surgery and the annual inflation rate using the sliders provided on the Graphical User Interface (GUI; Suppl. Fig. S1).

1.6 Submodels

1.6.1 update-age

Ages of all surviving dogs are updated (state variable '*aim*' – age in months) by 1 month during each time step.

1.6.2 update-immune-status

The immune status (state variable ‘*imm*’) of vaccinated dogs is updated each time step to represent waning immunity (see 2.6.6 for vaccination) . The duration of protective immunity post anti-rabies vaccination is assumed to be one year. In the developing world, a large proportion of the dog population is mostly free-roaming; these dogs are quasi-owned, never restrained nor subject to health or fertility control interventions. In such populations, there is a variation in antibody response to a single dose of anti-rabies vaccine, but most dogs do not have a protective antibody titer after a year [4].

1.6.3 follow-up-vaccination

A monthly probability derived from the follow-up vaccination rate (slider: *followup-vacc-rate*) determines if a neutered dog is revaccinated. Neutered dogs with duration of immunity 6 months or less (*imm* < 7, i.e. not revaccinated in the last 6 months) are revaccinated.

1.6.4 dog-mortality

Dog populations in the developing world have a high turnover rate (30-50%) [5–8]. The annual survival estimate for adult FRD is 70% [9]. Reece et al.[9] report a low survival rate (25%) for juvenile FRD. We use these estimates to derive monthly probabilities of death for adult and juvenile dogs in the model population. Additionally, the size of the adult dog population has a density-dependent effect on juvenile mortality, such that when adult population size reaches or exceeds adult dog carrying capacity, juvenile mortality is at its maximum (75% per year) [9]. As the abundance of adult dogs in the model population goes below the adult dog carrying capacity, juvenile survival increases. The effect of decreasing adult abundance on annual juvenile survival as modelled in DogPopDy is shown in Suppl. Figure 2.

Rates are converted into monthly probabilities using the following equation:

$p = 1 - \exp\{-rt\}$

where p=probability, r= instantaneous rate, provided that it is constant over the period of interest (t) [10].

1.6.5 dogs-reproduce

Reproduction is simulated over a six month period every year, between month 9 (September) and month 2 (February) [11,12]. As female dogs become sexually mature

earliest at 6 months of age, [13] and the gestation period is 2 months, we have assumed that females older than 8 months will breed (parturition) once during a breeding season. Each month of the breeding season, a proportion of intact adult and subadult (> 8 months old) females produce a litter. The proportion of intact females that reproduce during a breeding season is influenced by the ratio of intact adult males to intact adult females in the model dog population. Investigations of a roaming dog population in an Indian city indicated that 47.5% (95%CI 43 -51) of adult females reproduce annually [9]. The proportion of intact females that reproduce is therefore set between 0.44 and 0.51, if the intact male: intact female ratio in the model dog population is more than 0.1. If the ratio is lower than 0.1, we assume that the proportion of intact females that reproduce will decrease. In such a scenario, the number of pregnant females is calculated under the assumption that each intact male impregnates an average of 10 (range 3 – 17) females.

An average litter size of 5.6 was documented in urban, FRD, based on the number of aborted foetuses recorded during sterilization surgeries (ABC program) in Jaipur, India [14]. In this model, we have set the average litter size at 4 ± 0.5 SD to adjust for embryonic and neonatal losses. Sex ratio at birth is set at 1:1.

1.6.6 dog-sterilization

ABC is implemented by iterating over non-neutered dogs older than 6 months and changing the reproductive status of selected individuals to 'neutered'. The monthly target for ABC is limited by the number of ABC centers (user-specified, slider: *num-abc-centers*) and the maximum number of surgeries per ABC center. As per the guidelines formulated by the Animal Welfare Board of India (AWBI), the number of surgeries per month is capped at 250 per ABC facility[15].

For the best case scenario (closed population, all dogs are equally and easily catchable), stochasticity in the dog capturing process is modeled by decreasing the monthly target by up to 5%. We assume that the monthly target so derived is equally divided between males and females.

For the real-world scenario, we incorporate processes that affect dog captures, and therefore the actual number of ABC surgeries per month. Capture effort heterogeneity is an important factor influencing the number of dogs captured for the

ABC program. While planning dog population management strategies, it is often assumed that all dogs are catchable. However, in the real world, the capture effort, and therefore the capture efficiency, varies between dogs. Some dogs are easy to capture (cooperative reference persons, friendly community-owned dogs accustomed to handling), while others require additional effort and time (capture using nets, cages, chemical immobilization). Based on free-ranging dog capture data from rural as well as urban sites[16] (A. T. Vanak unpublished data), we estimated that 40% dogs in free-ranging dog populations are easy to capture while 60% require additional effort than what is normally deployed by the dog catching team. To incorporate capture effort heterogeneity in the model, we have included a dog state variable 'catchability'. A non-zero catchability between 1 and 100 is randomly assigned to each dog in the model population. Dogs with catchability > 60 have a capture probability of 1 (readily accessible with standard catch effort), dogs with catchability between 30 and 60 have a capture probability of 0.67 (require 50% more effort than standard catch effort), and dogs with catchability below 30 have a capture probability of 0.5 (require 100% more effort than standard catch effort). The proportion of unneutered dogs that are catchable during a time step is determined as follows:

Proportion of unneutered catchable dogs = {(Number of unneutered dogs with catchability > 60 * 1) + (Number of unneutered dogs with catchability between 30 and 60 * 0.67) + (Number of unneutered dogs with catchability < 30 * 0.5)} / Total dogs

The monthly ABC target is scaled using the proportion of unneutered catchable dogs.

For the real world scenario, it is also possible to designate a proportion of the dog population as inaccessible (using the slider '*piad*'). Owned, free-ranging dogs protected by their owners from the dog catching team or truly feral dogs fall in this category. Inaccessible dogs remain intact and unvaccinated throughout the simulation.

The desired target for the ABC program can be set using slider '*target-reduction*' (the desired reduction expressed as percent of the initial population). A post-target ABC rate can also be specified (set using slider: *followup-abc-rate*) – this rate is

implemented if the desired reduction in dog population is achieved before the ABC program duration is completed. All sterilized dogs are vaccinated against rabies, and the duration of immunity is set at 12 months.

1.6.7. dogs-immigrate

Immigration of dogs into the model dog population is simulated if the user-specified annual immigration rate is more than zero. This rate is interpreted in the context of the initial dog carrying capacity. The number of dogs immigrating during each month of the model run is determined using this rate. All immigrating dogs are sexually intact (non-neutered) and between one and two years of age. We assumed that male: female ratio in the immigrating dogs is set at 1:1.

Supplementary Table S1. State variables of agents included in DogPopDy.

**Agent**	**State variable**	**Meaning/Value**
Patches	*patch_dog_density*	Sets the initial number of dogs on a patch; derived from a normal distribution around average number of dogs per patch ± 20% SD. Average number of dogs per patch is calculated using the carrying capacity (human population / human-dog ratio) and total number of patches.
Dogs	*aim*	age in months
	sex	male = 1, female = 0
	bred?	TRUE for females bred in current year
	bc?	(birth control) TRUE if neutered, FALSE if intact
	ia	0 if readily accessible, 1 if inaccessible
	imm	protective immunity as a result of rabies vaccination;

	0: no protective immunity, 1 – 12: duration of protective immunity (months)
cap-p	capture probability between 1 and 100, both inclusive

Supplementary Figure S1. A screenshot of the model dashboard of DogPopDy in NetLogo with the various parameter adjustments and project simulation tools available, as well as the visual outputs of the simulation results.

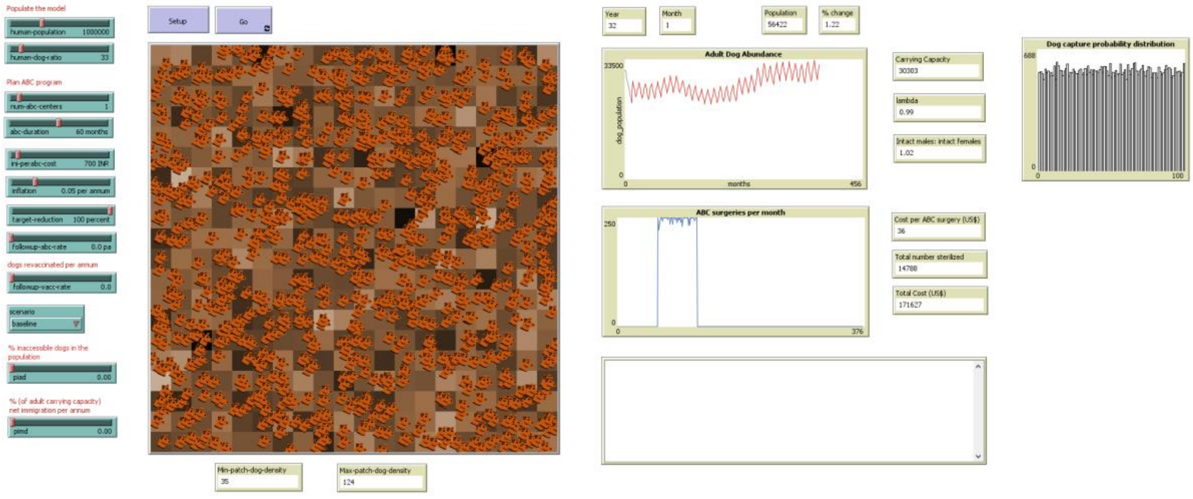

Supplementary Figure S2. Juvenile survival is modelled as a density-dependent process in DogPopDy such that only 25% juvenile dogs survive beyond the age of one year when adult dogs are at carrying capacity. Juvenile survival increases as adult abundance decreases, but the maximum rate of juvenile survival does not exceed 50%.

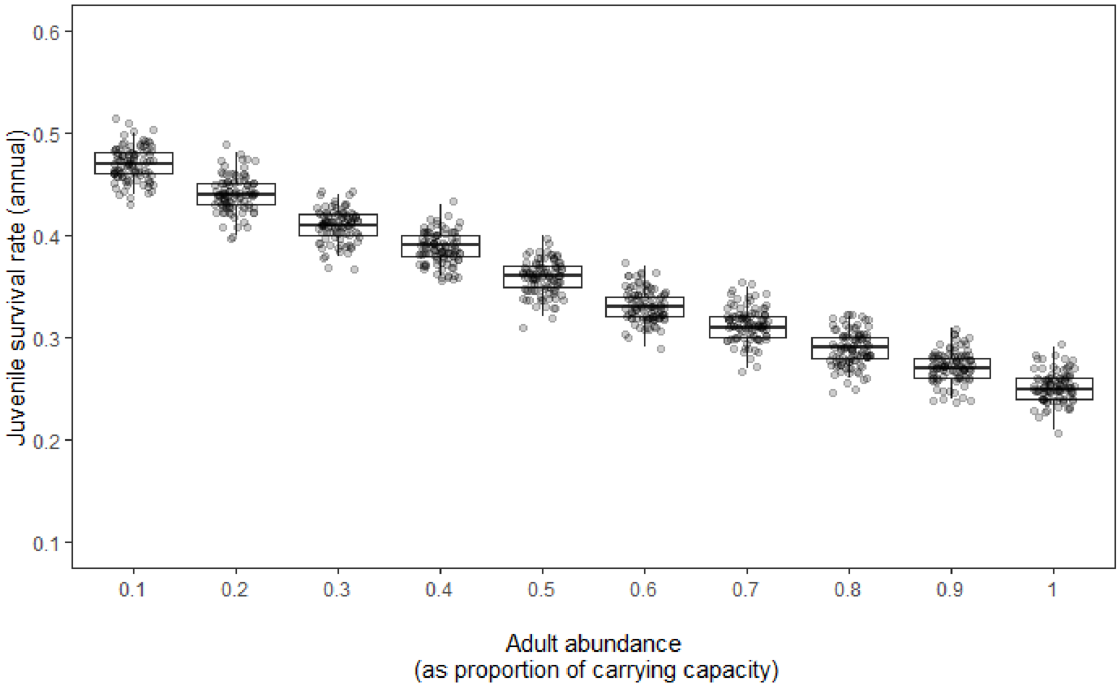

References

1. Wilensky, U. NetLogo, Center for Connected Learning and Computer-Based Modeling, Northwestern University, Evanston, IL. (1999).
2. Grimm, V. *et al.* A standard protocol for describing individual-based and agent-based models. *Ecol. Modell.* **198**, 115–126 (2006).
3. Grimm, V. *et al.* The ODD protocol: A review and first update. *Ecol. Modell.* **221**, 2760–2768 (2010).
4. Pimburage, R. M. S., Gunatilake, M., Wimalaratne, O., Balasuriya, A. & Perera, K. A. D. N. Sero-prevalence of virus neutralizing antibodies for rabies in different groups of dogs following vaccination. *BMC Vet. Res.* **13**, 1–10 (2017).
5. Matter, H. C. Canine ecology and rabies vaccination. *Symposium on Rabies control in Asia* 75–94 (1993).
6. Beran, G. Ecology of dogs in the Central Philippines in relation to rabies control efforts. *Comp. Immunol. Microbiol. Infect. Dis.* **5**, 265–70 (1982).
7. Kitala, P. *et al.* Dog ecology and demography information to support the planning of rabies control in Machakos District, Kenya. *Acta Trop.* **78**, (2001).
8. Belsare, A. V. & Gompper, M. E. A model-based approach for investigation and mitigation of disease spillover risks to wildlife: Dogs, foxes and canine distemper in central India. *Ecol. Modell.* **296**, 102–112 (2015).
9. Reece, J. F., Chawla, S. K., Hiby, E. F. & Hiby, L. R. Fecundity and longevity of roaming dogs in Jaipur, India. *BMC Vet. Res.* **4**, 6 (2008).
10. Briggs, A. H., Claxton, K. & Sculpher, M. J. *Decision Modelling for Health Economic Evaluation*. (OUP Oxford, 2006).
11. Pal, S. K. Population ecology of free-ranging urban dogs in West Bengal, India. *Acta Theriol. (Warsz).* **46**, 69–78 (2001).
12. Pal, S. K. Mating system of free-ranging dogs (Canis familiaris). *Int. J. Zool.* (2011) doi:10.1155/2011/314216.
13. Kustritz, M. V. R. *Clinical canine and feline reproduction: evidence-based answers*. (John Wiley & Sons, 2011).
14. Chawla, S. K. & Reece, J. F. Timing of oestrous and reproductive behaviour in Indian street dogs. *Vet. Rec.* **150**, 450–451 (2002).
15. AWBI. *Revised module for street dog population management, rabies eradication, reducing man-dog conflict*. http://awbi.org/awbi-pdf/revised_abc_module.pdf (2016).
16. Belsare, A. V. & Gompper, M. E. Assessing demographic and epidemiologic parameters of rural dog populations in India during mass vaccination campaigns. *Prev. Vet. Med.* **111**, 139–146 (2013).
